# Suppressive Effects of the Site 1 Protease (S1P) Inhibitor, PF-429242, on Dengue Virus Propagation

**DOI:** 10.3390/v8020046

**Published:** 2016-02-10

**Authors:** Leo Uchida, Shuzo Urata, Gianne Eduard L. Ulanday, Yuki Takamatsu, Jiro Yasuda, Kouichi Morita, Daisuke Hayasaka

**Affiliations:** 1 Department of Virology, Institute of Tropical Medicine, Nagasaki University, 1-12-4 Sakamoto, Nagasaki 852-8523, Japan; uchidaleo@rakuno.ac.jp (L.U.); ulanday@tm.nagasaki-u.ac.jp (G.E.L.U.); yuki.takamatsu@staff.uni-marburg.de (Y.T.); moritak@nagasaki-u.ac.jp (K.M.); hayasaka@nagasaki-u.ac.jp (D.H.); 2 Department of Emerging Infectious Disease, Institute of Tropical Medicine, Nagasaki University, 1-12-4 Sakamoto, Nagasaki 852-8523, Japan; shuzourata@nagasaki-u.ac.jp (S.U.); j-yasuda@nagasaki-u.ac.jp (J.Y.); 3 Graduate school of Biomedical Sciences, Nagasaki University, 1-12-4 Sakamoto, Nagasaki 852-8523, Japan; 4 Leading graduate school program, Nagasaki University, 1-12-4 Sakamoto, Nagasaki, 852-8523, Japan

**Keywords:** dengue virus, antivirus drug, PF-429242, SREBPs, S1P

## Abstract

Dengue virus (DENV) infection causes one of the most widespread mosquito-borne diseases in the world. Despite the great need, effective vaccines and practical antiviral therapies are still under development. Intracellular lipid levels are regulated by sterol regulatory elements-binding proteins (SREBPs), which are activated by serine protease, site 1 protease (S1P). Small compound PF-429242 is known as a S1P inhibitor and the antivirus effects have been reported in some viruses. In this study, we examined the anti-DENV effects of PF-429242 using all four serotypes of DENV by several primate-derived cell lines. Moreover, emergence of drug-resistant DENV mutants was assessed by sequential passages with the drug. DENV dependency on intracellular lipids during their infection was also evaluated by adding extracellular lipids. The addition of PF-429242 showed suppression of viral propagation in all DENV serotypes. We showed that drug-resistant DENV mutants are unlikely to emerge after five times sequential passages through treatment with PF-429242. Although the levels of intracellular cholesterol and lipid droplets were reduced by PF-429242, viral propagations were not recovered by addition of exogenous cholesterol or fatty acids, indicating that the reduction of LD and cholesterol caused by PF-429242 treatment is not related to its mechanism of action against DENV propagation. Our results suggest that PF-429242 is a promising candidate for an anti-DENV agent.

## 1. Introduction

Dengue is one of the most widespread mosquito-borne diseases in the world. Recent studies estimated that there are 390 million Dengue cases in tropical and sub-tropical regions annually [1]. The causative agent, dengue virus (DENV), belongs to the family *Flaviviridae*, genus *Flavivirus,* and includes four main serotypes (DENV1 to 4). Its viral genome is a single positive strand RNA encoding three structural (C, prM and E) and seven non-structural (NS1, NS2A, NS2B, NS3, NS4A, NS4B and NS5) proteins.

DENV infection causes a range of symptoms, from mild febrile illness (Dengue fever) to severe hemorrhagic fever (Dengue hemorrhagic fever and Dengue shock syndrome). However, effective commercial vaccines and practical antiviral therapies are still under development [2,3]. It is suggested that a secondary DENV infection, caused by a different serotype from the primary infection, is one of the risk factors for the severity of the disease [4,5]. This disease mechanism makes it more difficult to develop a tetravalent DENV vaccine; thus, the development of an effective treatment against all serotypes of DENV is an important priority.

DENV utilizes biological lipids, such as cholesterol, triglycerides and phospholipids, for replication in infected cells. For example, it has been suggested that intracellular lipids, such as cholesterol-rich membrane and lipid droplets (LD), play important roles in DENV entry [6] and in the localization of viral capsid (C) proteins [7]. It has also been shown that cholesterol depletion agents, such as methyl-β-cyclodextrin, and hypolipidemic agents, such as lovastatin, inhibit DENV infection *in vitro* [8,9]. Therefore, it is expected that the intracellular lipids are potential targets for the development of DENV treatments.

The small molecule PF-429242 was developed as a hypolipidemic agent based on high throughput screening in a Pfizer compound library [10,11]. A broad spectrum of intracellular lipids is regulated by transcriptional regulators, including sterol regulatory elements-binding proteins (SREBPs) [12]. The SREBPs positively regulate some lipogenic genes, such as 3-hydroxy-3-methylglutaryl-coenzyme A (HMG-CoA) synthase, HMG-CoA reductase, and squalene synthase [12]. The immature SREBPs are cleaved by two proteases; site-1 protease (S1P) (also known as subtilisin kexin isozyme-1, SKI-1) and site-2 protease. The released basic helix-loop-helix–leucine zipper (bHLH-Zip) domain on the N-terminal side of SREBPs translocates into the nucleus and regulates sterol response elements (SREs) as a transcription factor [13,14]. PF-429242 inhibits the activity of S1P reversibly and competitively and suppresses the expression level of SREBP target genes, consequently decreasing cellular lipid levels [11]. It has been shown that PF-429242 suppresses hepatic SREBP target genes and inhibits cholesterol and fatty acid synthesis in a mouse model [11].

Recently, it has been reported that PF-429242 suppresses viral replication in cells infected with hepatitis C virus (HCV), Lassa virus, lymphocytic choriomeningitis virus, and New World arenaviruses. [15,16,17,18,19]. These observations potentially provide a possible approach for the effective treatment of DENV infection. Therefore, in this study, we investigated whether PF-429242 has an antiviral effect on DENV infection and replication using cultured cells.

## 2. Materials and Methods

### 2.1. Cells and Viruses

Human epithelial HeLa, HEK-293, Hep G2, non-human primate epithelial LLC-MK2, and mosquito-derived C6/36 E2 cells were maintained in minimum essential medium (MEM) supplemented with 10% fetal calf serum (FCS) and 0.2 mM nonessential amino acids (aa.). The cells were allowed to grow at 37 °C with 5% CO_2_ for mammalian cells and at 28 °C without CO_2_ for C6/36 E2 cells. A tissue-culture adapted DENV1 strain from Hawaii, the infectious cloned-derived DENV2 strain 16681 (GenBank: U87411) [20], and patient-derived strains DENV3 SLMC50 and DENV4 SLMC318 from the Philippines were used for this study. Viruses were propagated in C6/36 E2 cells to generate working stocks, and the viral titer was determined by focus-forming assay (FFA) using C6/36 E2 cells.

### 2.2. Chemical Reagents

Ten milligrams of compound PF-429242 (TOCRIS bioscience, Avonmouth, Bristol, UK) were dissolved in 1.96 mL and 196 µL of dimethyl sulfoxide (DMSO) (Sigma, St. Louis, MO, USA) to make 10 mM and 100 mM stock solutions, respectively. The aliquots were kept at −30 °C until use in the experiments. Cholesterol lipid concentrate (Life technologies, Waltham, MA, USA) was used to restore the intracellular cholesterol. The amount of original cholesterol lipid concentrate was evaluated by Amplex Red Cholesterol Assay kit (Invitrogen, Grand Island, NY, USA) according to the methods described in the instruction manual. A fatty acid, sodium oleate, (Sigma) was used to restore the intracellular LD [21]. Two hundred fifty milligrams of sodium oleate were dissolved in 4.1 mL of deionized-distilled water to make a 200 mM stock solution, and the aliquots were kept at 4 °C until the experiments started.

### 2.3. DENV Propagation with PF-429242

The HeLa, HEK-293, Hep G2, and LLC-MK2 cells were prepared in 12 or 24-well plates (Cat No. 150628 or 142475, Thermo Fisher Scientific, Waltham, MA, USA) at 80% to 90% confluence. The indicated multiplicity of infection (MOI) of the DENV was prepared in the culture medium containing prescribed concentrations of PF-429242. The same volume of the DMSO was used as negative control. The final concentration of DMSO is less than 0.3% in the medium. The mixture was introduced to the cells and maintained at 37 °C with 5% CO_2_. At the indicated time post-infection (p.i.), culture fluid and/or cells were collected for virus titration by FFA and real-time quantitative reverse transcription PCR (RT-qPCR). In other studies, 5 to 500 µg/mL of the cholesterol lipid concentrate, or 2 to 20 µM of the sodium oleate stock, were added at the same time as the drug application to restore the intracellular cholesterol and LD. The DENV titer in all of the culture fluids and the cells were evaluated by FFA and RT-qPCR, in accordance with our previous study [22].

### 2.4. Cytotoxicity Assay

HeLa cells were seeded in MEM supplemented with 2% FCS and 0.2 mM nonessential amino acids, which contained 3 to 300 µM of PF-429242. After 72 h post drug treatment, a CellTiter-Glo (Promega, Madison, WI, USA) mixture was added to the cultured cells, and the intracellular ATP was measured for luminescence intensity with ARVO MX/Light 1420 Multilabel/Luminescence counter (Perkin Elmer, Waltham, MA, USA).

### 2.5. Escape Mutant Study

Before virus infection, the viral RNA (vRNA) copy numbers corresponding to MOI 0.5 were calculated by RT-qPCR, as described above [22]. HEK-293 cells were infected with the DENV2 strain 16681 at MOI 0.5 in the medium, which contained 6 µM of PF-429242 or the same amount of DMSO. At 96 h p.i., the supernatant was collected, and the vRNA was quantified by RT-qPCR. The harvested-DENV corresponding to the MOI 0.5 was used to continuously infect the next HEK-293 cells and so forth. A total of 5 sequential passages were conducted.

### 2.6. Quantification of Intracellular Lipids

The levels of intracellular cholesterol were quantified by an Amplex Red Cholesterol Assay kit (Invitrogen) according to the manuscripts [15]. Briefly, the cells in the 12-well plates were washed with phosphate-buffered saline (PBS) and lysed in 500 µL of 1% of polyoxyethylene (10) octylphenyl ether (Triton X-100) in PBS [15]. The cell lysate was diluted 10-fold with a kit-derived reaction buffer. Then, 50 µL of the diluted lysate and 50 µL of the Amplex Red solution were mixed and followed by incubation at 37 °C for 30 min. In another experiment, the cells in the 96-well plate were fixed with 4% paraformaldehyde (PFA) and the intracellular LD was stained with 4,4-difluoro-1,3,5,7,8-pentamethyl-4-bora-3a,4a-diaza-s-indacene (BODIPY 493/503) (Thermo Fisher Scientific). In both experiments, the intensity of the fluorescence was measured with an ARVO MX/Light according to the following conditions: resorufin for cholesterol assay, excitation (Ex); 485 nm, emission (Em); 590 nm, and BODIPY 493/503 for LD assay, Ex: 485 nm, Em 515 nm, respectively.

### 2.7. Transient Expression of rC and rNS2B/3

DENV2 16681 strains coding for the C (aa. 1–114) and NS2B/3 complexes (aa. 1346–2093) were cloned in mammalian-expressing plasmid pcDNA3.1(+) (Invitrogen). The x3 FLAG-tag fragment was inserted into the C-terminal side of the target sequence. The plasmids were transfected into HeLa cells by Microporator MP-100 (Digital Bio, Seoul, Korea) according to the following settings: pulse voltage, 1005 v; pulse width, 35 ms; pulse no., 2. The transfected cells were allowed to grow at 37 °C with 5% CO_2_. DENV infected, uninfected, or plasmid-transfected HeLa cells were prepared in 8-well Millicell EZ slides (Millipore, Darmstadt, Germany).

### 2.8. Immunofluorescence Assay

At 48 h after transfection with recombinant complementary DNA (cDNA) plasmids containing DENV proteins, cells were fixed with 4% PFA containing 4% sucrose for 20 min and permeabilized by 0.1% Triton X-100 for 4 min at room temperature [7]. Anti-FLAG M2 (Sigma), anti-double stranded-RNA (dsRNA) K1 (English & Scientific Consulting Kft., Szirák, Hungary) and 12D11/7E8 monoclonal Ab [23,24] were used to visualize the rC, rNS2B/3, viral dsRNA and E proteins, respectively. The primary antibodies were detected by Alexa Fluor 594-conjugated secondary antibodies (Invitrogen) with 1 µg/mL of BODIPY 493/503. The stained cells were sealed with ImmunoSelect Antifading Mounting Medium (Dianova GmbH, Hamburg, Germany), which contained 4’, 6-Diamidino-2-phenylindole (DAPI). Images were captured with a LSM 780 confocal laser-scanning microscope (Carl Zeiss, Oberkochen, Germany).

### 2.9. Statistical Analysis

A Welch test analysis was used to assess significant differences in viral titers, viral RNA levels, and cholesterol and LD abundances.

## 3. Results

### 3.1. Suppressive Effects of PF-429242 on DENV Replication

**Figure 1:**
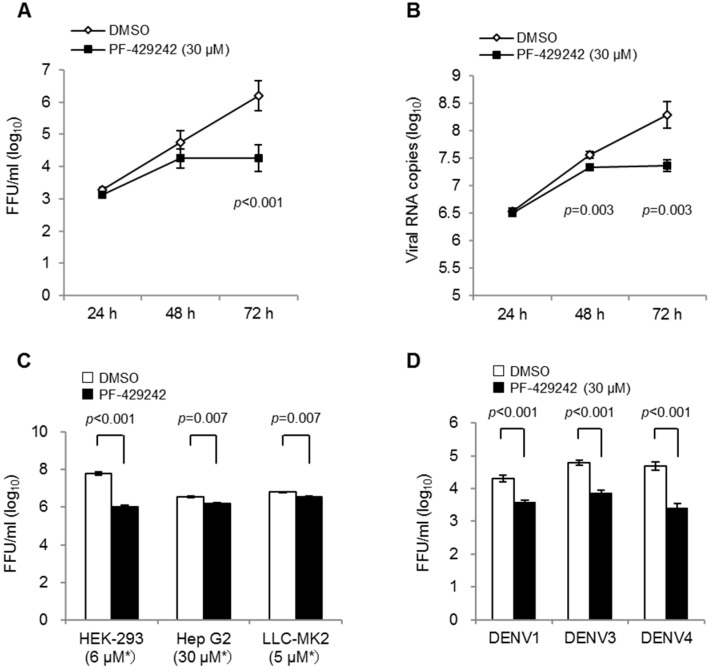
DENV suppression via the addition of PF-429242. (**A,B**) HeLa cells were infected with the DENV2 16681 strain at MOI 1 containing 30 μM of PF-429242 or DMSO alone (control) in the medium. Infectious viral titers (**A**) and viral RNA copy numbers (**B**) in the culture fluids at 24, 48 and 72 h were determined by focus-forming assay and real-time RT-PCR, respectively; (**C**) Infectious viral titers in the cell culture fluids of several cells at 72 h p.i. HEK-293, Hep G2, and LLC-MK2 cells were infected with DENV2 16681 strain at MOI 1 containing 6 μM, 30 μM, and 5 μM of PF-429242 respectively or DMSO only in the medium. Asterisks indicate the concentration of PF-429242; (**D**) Infectious viral titers in the cell-culture fluids of HeLa cells at 72 h p.i. HeLa cells were infected with DENV1 strain from Hawaii, DENV3 SLMC50, and DENV4 SLMC318 at MOI 1 containing 30 μM of PF-429242 or DMSO only in the medium. FFU: focus formation unit. Error bars indicate the standard error (SE). *p* values: Welch test analysis. Means and S.E. are obtained from 3 of duplicate experiments.

We first examined the suppressive effects of PF-429242 on DENV2 (16681 strain) propagation in human-derived epithelial HeLa cells. The addition of PF-429242 (30 μM) showed statistically significant suppression of infectious viral titers and viral RNA copies in the cell culture fluids compared with the control (DMSO alone)-treated cells at 72 h p.i (Figure 1A,B). We also showed the suppressive effects of PF-429242 on DENV2 yields in the cultured fluids of human-derived HEK-293, Hep G2, and non-human-primate derived LLC-MK2 cells (Figure 1C). Suppressive effects of PF-429242 on viral propagation were also observed in HeLa cells infected with other DENV serotypes: DENV1 (Hawaii strain), DENV3 (SLMC50 strain) and DENV4 (SLMC318 strain) (Figure 1D). The addition of PF-429242 in the cell lines mentioned did not show any noticeable alteration of cell morphology as observed via light microscopy (data not shown).

These results indicate that PF-429242 exerted suppressive effects on DENV replication. Furthermore, it is likely that PF-429242 effected all four serotypes of DENV and acted in some primate-derived cell lines.

### 3.2. Cytotoxic and Inhibitory Concentrations of PF-429242

**Figure 2:**
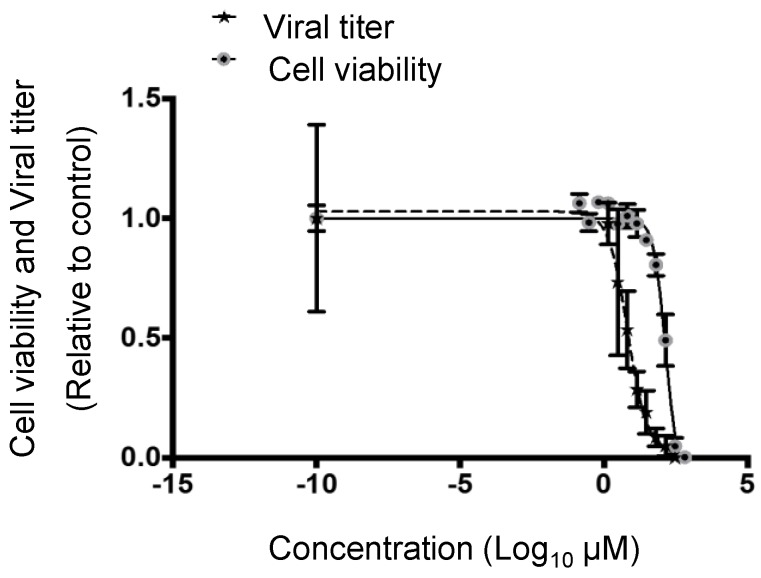
Cytotoxicity and inhibitory concentrations of PF-429242 in HeLa cells infected with DENV. HeLa cells were treated with 3 to 300 µM of PF-429242, and intracellular ATP was measured after 72 h. HeLa cells were infected with the DENV2 16681 strain at MOI 0.001 containing 1.4 to 300 μM of PF-429242 or DMSO alone in the medium, and infectious viral titers in the supernatant were measured after 72 h. The X-axis indicates the concentrations of PF-429242 and the Y-axis indicates the relative values compared with the control. Error bars indicate the S.E. Means and SE are obtained from 3 of duplicate experiments.

The addition of 300 µM of PF-429242 induced significant cytotoxicity in HeLa cells at 72 h p.i., and the 50% cytotoxic concentration (CC_50_) of PF-429242 was 236.7 µM (95% Confidence interval (CI): 87.6 µM to 639.2 µM) (Figure 2). On the other hand, the 50% inhibitory concentration (IC_50_) of PF-429242 against DENV yields in cell-culture fluids was 6.7 µM (95% CI: 4.0 µM to 11.2 µM), and the 90% inhibitory concentration was 35.5 µM (95% CI: 12.0 µM to 106.2 µM) (Figure 2). Thus, the IC_50_ and IC_90_ were respectively approximately 35 times and 6.7 times higher than the CC_50_.

### 3.3. Emergence of Drug-Resistant Mutants

**Figure 3:**
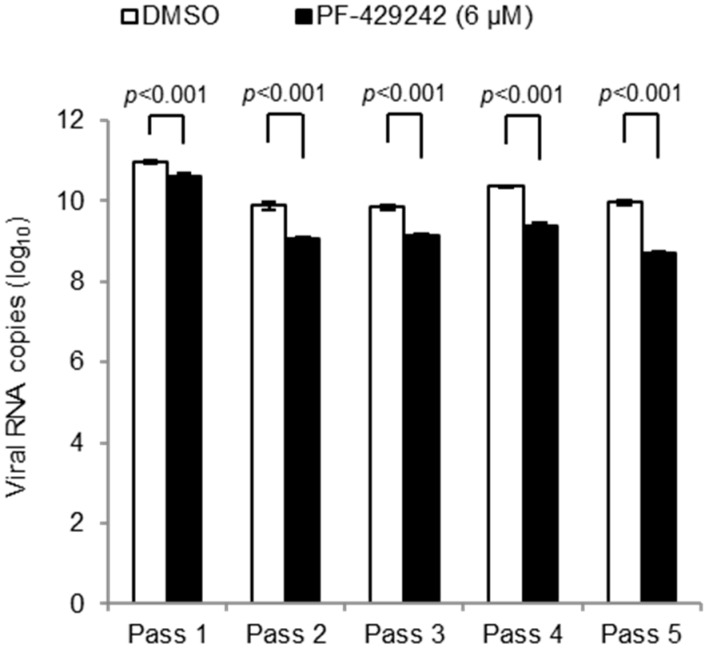
Viral titers in the culture fluids of DENV-infected cells during serial passages. HEK-293 cells were infected with DENV2 16681 strain at MOI 0.5 (4.58 × 10^8^ viral RNA copies/well) containing with 6 μM of PF-429242 or DMSO alone. Viral RNA copy numbers in the supernatant at 96 h p.i. were determined by real-time RT-PCR; 4.58 × 10^8^ viral RNA copies/wells of DENV were passed to fresh HEK-293 cells containing PF-429242 or DMSO. This procedure was repeated four times (for a total of five passages). Error bars indicate the SE. *p* values: Welch test analysis. Means and S.E. are obtained from single of sixtuplate experiments.

We next examined whether drug-resistant mutants appeared and constituted the major population of DENV progenitors during PF-429242 treatment. Virus yields in the cell-culture fluids undergoing PF-429242-treatment were passaged five times, and their viral loads were compared with those of mock-treatment. DENV titers in the culture fluids were significantly lower in PF-429242-treated cells than in mock-treated cells over five passages (Figure 3). These results indicate that drug-resistant DENV mutants did not appear to emerge after serial passages.

### 3.4. Intracellular Cholesterol Levels

**Figure 4:**
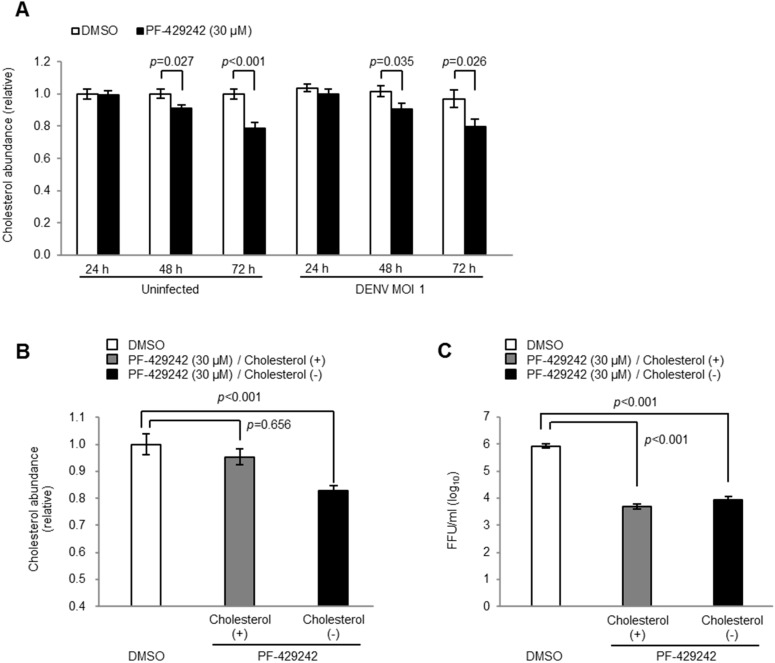
Intracellular cholesterol in DENV-infected cells. (**A**) Accumulation of intracellular cholesterol at 24, 48 and 72 h p.i. in HeLa cells infected with DENV2 16681 strain at MOI 1 containing 30 μM of PF-429242 or DMSO alone (**B** and **C**) Accumulation of intracellular cholesterol (**B**) and infectious viral titers in the supernatant (**C**) of DENV-infected cells at 72 h p.i. after the addition of exogenous cholesterol (500 µg/mL). FFU: focus formation unit. Error bars indicate the SE. Means and S.E. are obtained from 3 of duplicate experiments.

The addition of PF-429242 reduced the levels of intracellular cholesterol in both the mock-infected and DENV-infected cells (Figure 4A). Thus, to assess the influence of the reduced levels of intracellular cholesterol on DENV replication, exogenous cholesterols were added and the viral yields examined. The addition of cholesterol (500 µg/mL) in PF-429242-treated cells recovered the intracellular cholesterol levels to up to 95.3% of the mock-treated cells (Figure 4B). However, viral titers of PF-429242-treated cells infected with DENV were not recovered by additional cholesterol and were still significantly lower than mock-treated cells (Figure 4C). These observations suggest that intracellular cholesterol did not affect the DENV yields in the culture fluids of PF-429242-treated cells and that the reduced levels of intracellular cholesterol triggered by PF-429242 do not appear to contribute to its suppressive effects on DENV replication.

### 3.5. Intracellular LD Levels

**Figure 5:**
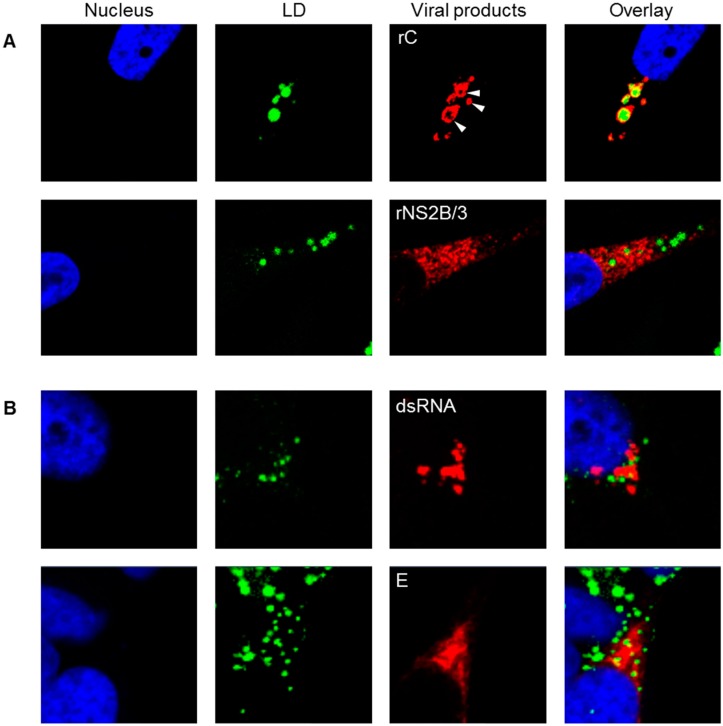
Localization of LD, viral RNA and proteins. (**A**) Expression plasmids containing the cDNA of DENV2 C and NS2B/3 proteins were transiently transfected into HeLa cells. Recombinant proteins were detected with anti-FLAG M2 antibody 48 h after transfection (**B**) HeLa cells were infected with DENV2 16681 strain at MOI 1. Viral dsRNA and E proteins were detected at 48 h p.i. using anti-dsRNA K1 Ab and 12D11/7E8 monoclonal antibodies, respectively. The cellular nucleus and intracellular LD were stained with DAPI and BODIPY 493/503, respectively. Blue, green, and red fluorescence colors indicate the nucleus, LD, and viral products, respectively. White arrow head indicates C proteins that accumulated around LD.

DENV C proteins accumulated around LD and constructed a “ring-shaped” structure, whereas other viral components, such as dsRNA, E and NS2B/3 proteins, were not morphologically related to LD (Figure 5). This raised the possibility that the reduced levels of LD exerted by PF-429242 contributed to its suppressive effect on DENV replication. Thus, we next examined the effects of LD abundance on DENV propagation.

**Figure 6:**
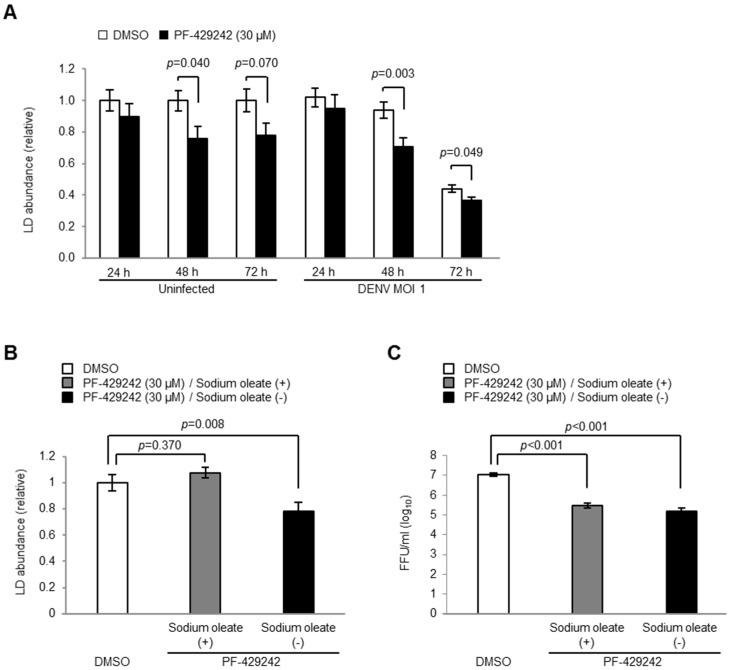
LD in DENV-infected cells. (**A**) Accumulation of LD at 24, 48 and 72 h p.i. in HeLa cells infected with DENV2 16681 strain at MOI 1 containing 30 μM of PF-429242 or DMSO alone (**B** and **C**) Accumulation of LD (**B**) and infectious viral titers in the supernatant (**C**) of DENV-infected cells at 72 h p.i. after the addition of exogenous oleic acid (20 µM). FFU: focus formation unit. Error bars indicate the SE. Means and S.E. are obtained from 3 duplicate experiments.

The addition of PF-429242 reduced the LD levels in both the DENV-infected and mock-infected cells (Figure 6A), *although s*ignificant morphological changes of the localization of C protein and LD were not observed by microscopy (data not shown). Thus, we next examined whether the reduced levels of LD contribute to the suppression of DENV replication. The addition of exogenous fatty acid sodium oleate (20 µM) recovered the LD levels of up to 107.5% of the mock-treated cells (Figure 6B). However, the addition of fatty acids did not affect the DENV yields in the cultured fluids of PF-429242-treated cells, and the viral loads were still significantly lower than those of mock-treated cells (Figure 6C).

These results suggest that the reduced levels of LD triggered by PF-429242 did not appear to contribute to its suppressive effects on DENV replication.

## 4. Discussion

In this study, we showed a suppressive effect of PF-429242 on DENV propagation in cultured cells. This inhibitory effect of PF-429242 was observed in several primate-derived cell lines infected with DENV2. PF-429242 also exerts suppressive effects on the viral replication of four serotypes of DENV1-4. Furthermore, we showed that drug-resistant DENV mutants are not likely to emerge after serial passages.

The anti-viral effects of PF-429242 have been reported in some arenaviruses [18,19]. Arenavirus glycoprotein (GP) precursor is cleaved into GP1 and GP2 by S1P, and this S1P-mediated cleavage is necessary for infectious virus production and its propagation. The disruption of S1P activity by PF-429242 results in a missed cleavage, and the virus propagation is suppressed [18,19].

On the other hand, there is no evidence that S1P is involved in the processing steps of the Flavivirus polyprotein, and the direct functions of S1P in DENV infection have not been elucidated. The Flavivirus-coding NS2B/3 complex shows serine protease activity. Thus, we examined whether PF-429242 inhibits the activity of NS2B/3 serine protease (NS2B/3pro) by using a recombinant protein-based *in vitro* assay. However, no inhibitory effects of PF-429242 on DENV NS2B/3pro activity were observed (Supplementary data Figure S1).

Physical interactions between the C proteins and LD have been reported in DENV and HCV infected-cells [7,25,26,27]. Marcelo M. Samsa *et al.* [7] suggested that two specific hydrophobic amino acids, L50 and L54, in the center of the C protein are necessary to interact with LD, and that the disruption mutants of these amino acids show lower or abortive virus production. On the other hand, Ivo C. Martins *et al.* [28] have shown that an interaction between positively charged N-terminal region of C protein and negatively charged LDs. Furthermore, it is suggested that the DENV C protein-LD interaction might be mediated by host perilipin 3 (TIP47) molecules on the surface of LD [25]. We also showed that DENV2 C proteins accumulated around LD.

Thus, we raised the initial possibility that quantitative and qualitative depletion of the intracellular LD might be the primary cause of the inhibition of DENV infection. However, although LD and cholesterol levels recovered through the addition of exogenous fatty acids and cholesterol, virus propagation was not restored completely. Previous studies have shown that the inhibition of S1P results in the suppression of HCV titers, but the supplementing of exogenous lipids, such as mevalonate, oleate, and cholesterol, result in an incomplete rescue of HCV propagation [15]. Taken together, our findings suggest that, although physical interactions between Flavivirus C proteins and LD or LD surface proteins appear to be necessary for virus propagation, the depletion of LD and cholesterol are not direct causes of the virus inhibition derived from PF-429242.

Other mechanisms may contribute to the suppressive effect of PF-429242 on DENV propagation. For example, Rothwell C. *et al.* [29] have reported that knockdown of mevalonate diphospho decarboxylase (MVD) inhibited DENV replication and that the MVD is positively regulated by SREBP-2. Thus, not only cellular lipids but also host proteins related to lipid metabolism may be relevant to the inhibitory mechanism of PF-429242. Furthermore, the inhibitory effects on DENV propagation were observed at the late stage of the infection (48 to 72 h p.i.) but not at the early stage (24 h p.i.), indicating that PF-429242 may not target virus polyprotein directly, but affects host cellular factors which play roles in the virus infection. Inhibitory effects of PF-429242 on DENV propagations were higher in HeLa cells and HEK-293 cells than Hep G2 cells and LLC-MK2 cells. Thus, the drug susceptibility may be related with the expression levels of such host cellular factors between different cell lines.

In this study, we observed that intracellular LD levels were decreased in DENV-infected cells compared with mock-infected cells at late stage of the infection (72 h p.i.). LD is one of the intracellular energy source mainly consisted of triacylglycerol, cholesterol ester, and some associated proteins. The relationships between LD and viral replication have been reported for HCV [15,16,26], DENV [7], Rotavirus [30], and Orthoreovirus [31]. These studies suggested that LD is vital in viral replication and assembling; however, quantitative and qualitative change of LD itself during virus infection remains to be elucidated. LD degradation has been reported in HCV replicon study [32]. For this reason, Vogt D. A. *et al.* [32] suggested that “LDs are being consumed during HCV replication, as a potential source for membranes or energy”. Thus, LD levels might be decreased in DENV-infected cells as much energy is required for virus replication.

Although the mechanism of the inhibitory effects of PF-429242 on DENV propagation remains uncertain, the SREBPs, whose function is modulated by S1P, might be an attractive drug target for DENV infection. Thus, PF-429242 is expected be a potential candidate for an anti-DENV agent. Further study focusing on the inhibitory mechanisms of PF-429242 will provide novel clues for the development of an effective anti-DENV treatment.

## 5. Conclusions

In this study, we examined anti- DENV effects of PF-429242, a potent inhibitor of site 1 protease (S1P). The addition of PF-429242 suppressed the viral propagation of all serotypes of DENV in several primate-derived cells. In our experiments, drug-resistant DENV mutants are unlikely to emerge. Although the levels of intracellular lipids were reduced by PF-429242, viral propagations were not recovered by exogenous cholesterol or fatty acids, indicating that these depletions of LD and cholesterol do not explain the anti-DENV effects of PF-429242 treatment. Here we showed an evidence that PF-429242 could be a potential candidate for an anti-DENV drug, although the mechanism of the inhibitory effects are still unclear.

## Supplementary Materials

Figure S1: Recombinant protein-based *in vitro* assay of DENV NS2B/3pro.
