# Supplementary material for: Suppressive Effects of the Site 1 Protease (S1P) Inhibitor, PF-429242, on Dengue Virus Propagation
Source: Viruses. 2016 Feb 10;8(2):46. doi: 10.3390/v8020046 (PMC4776201; doi:10.3390/v8020046)
Supplement: Supplementary file 1 [file viruses-08-00046-s001.pdf]

viruses

Article

Suppressive Effects of the Site 1 Protease (S1P) Inhibitor, PF-429242, on Dengue Virus Propagation

Leo Uchida [1], Shuzo Urata [2], Gianne Eduard L. Ulanday [1,3], Yuki Takamatsu [1], Jiro Yasuda [2,4], Kouichi Morita [1,4] and Daisuke Hayasaka [1,4,*]

Supplementary Material

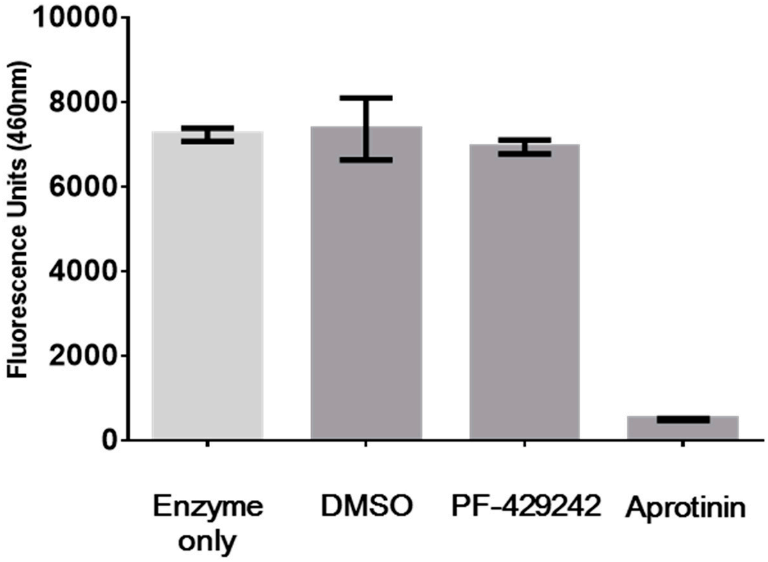

Figure S1. Recombinant protein-based in vitro assay of DENV NS2B/3pro. 100 μM each of PF-429242 and DENV NS2B/3pro control inhibitor, Aprotinin, were mixed in separate wells containing 1 μg/ml of recombinant DENV NS2B/3pro. Mixture was incubated at 25℃ for 30 minutes after the addition of 100 μM fluorescence-labeled substrate. Fluorescence intensity was measured using ARVO MX/Light 1420 Multilabel / Luminescence counter (Perkin Elmer) at 460 nm. Means are obtained from 2 of duplicate experiments. Error bars show standard deviation.

© 2016 by the authors; licensee MDPI, Basel, Switzerland. This article is an open access article distributed under the terms and conditions of the Creative Commons by Attribution (CC-BY) license (http://creativecommons.org/licenses/by/4.0/).
